# Quantification of Costal Cartilage Calcification Using ^18^F-NaF-PET/CT

**DOI:** 10.3390/jimaging12050206

**Published:** 2026-05-12

**Authors:** Vanessa Shehu, Om H. Gandhi, Patrick Glennan, Jaskeerat Gujral, Shashi B. Singh, Amir A. Amanullah, Shiv Patil, Khushi Gujral, William Y. Raynor, Peter Sang Uk Park, Eric M. Teichner, Robert C. Subtirelu, Talha Khan, Thomas J. Werner, Poul Flemming Høilund-Carlsen, Ali Gholamrezanezhad, Mona-Elisabeth Revheim, Abass Alavi

**Affiliations:** 1 Department of Radiology, Hospital of the University of Pennsylvania, Philadelphia, PA 19104, USA; vas194@pitt.edu (V.S.); omgandhi@sas.upenn.edu (O.H.G.); pglennan@sas.upenn.edu (P.G.); jgujral@sas.upenn.edu (J.G.); shiv.patil@students.jefferson.edu (S.P.); khushigujral05@gmail.com (K.G.); peter.park@pennmedicine.upenn.edu (P.S.U.P.); robert.subtirelu@pennmedicine.upenn.edu (R.C.S.); talha_khan@med.unc.edu (T.K.); tom.werner@pennmedicine.upenn.edu (T.J.W.); 2 School of Medicine, University of Pittsburgh, Pittsburgh, PA 15213, USA; 3 Department of Nuclear Medicine, Odense University Hospital, 5000 Odense, Denmark; pfhc@rsyd.dk; 4 Department of Clinical Research, University of Southern Denmark, 5000 Odense, Denmark; 5 Department of Radiology, Keck School of Medicine, University of Southern California, Los Angeles, CA 90033, USA; agholamrezanezhad@health.ucsd.edu; 6 The Intervention Center, Division for Technology and Innovation, Oslo University Hospital, 0424 Oslo, Norway; monar@ous-hf.no; 7 Institute of Clinical Medicine, Faculty of Medicine, University of Oslo, 0313 Oslo, Norway

**Keywords:** costal cartilage, positron emission tomography/computed tomography (PET/CT), ^18^F-sodium fluoride (^18^F-NaF), calcification, aging

## Abstract

A quantification technique for costal cartilage calcification using ^18^F-sodium fluoride–positron emission tomography/computed tomography (^18^F-NaF-PET/CT) has yet to be established, and the effects of aging and other demographic variables on costal cartilage calcification remain understudied. This study aims to introduce a quantification methodology for assessing costal cartilage calcification using ^18^F-NaF-PET/CT, assess age-related changes in its ^18^F-NaF uptake in females and males, and examine the relationship between its ^18^F-NaF uptake and CT attenuation as well as ^18^F-NaF uptake and coronary artery calcification. In this retrospective study, we analyzed subjects from the Cardiovascular Molecular Calcification Assessed by ^18^F-NaF PET/CT (CAMONA) clinical trial. This study evaluated 130 subjects (mean age 48.7 ± 14.5 years; n = 67 females). We manually generated regions of interest overlying the costal cartilages from ribs 8 to 10 on the left side, carefully avoiding osseous uptake from adjacent ribs and sternum, to measure cartilaginous ^18^F-NaF uptake. Non-parametric statistical analyses (Spearman correlations, Mann–Whitney U tests, Kruskal–Wallis tests) and receiver operating characteristic analysis were performed to evaluate sex-specific age-related changes in uptake, correlations between imaging parameters, and associations with coronary artery calcium (CAC) score. In females, the mean ^18^F-NaF uptake (as assessed by average SUV_mean_) was 0.69 ± 0.38 while the corresponding mean Hounsfield Unit (HU) was 108.0 ± 40.0. In males, the mean ^18^F-NaF uptake (as assessed by average SUV_mean_) was 0.63 ± 0.22, and the mean HU was 104.0 ± 24.0. There was a significant correlation between ^18^F-NaF uptake and age in both females (*p* = 0.003, r = 0.36) and males (*p* < 0.0001, r = 0.63). The correlation was significantly stronger in males than females (Fisher’s z-test, *p* = 0.040). There was a significant correlation between CAC score and costal cartilage SUV_mean_ in both females (r = 0.26, *p* = 0.036) and males (r = 0.51, *p* < 0.0001). This study introduces a quantification technique to assess costal cartilage calcification using ^18^F-NaF-PET/CT and demonstrates that the calcification increases with age, more strongly in males than in females, and ^18^F-NaF uptake is correlated with CAC score. This technique can be applied to other cartilages of interest, in both physiological and pathological conditions, to assess the effects of aging and various demographic variables on cartilage calcification.

## 1. Introduction

Costal cartilage is a type of hyaline cartilage that provides structural support for the thoracic cavity and facilitates chest wall movement during breathing. While costal cartilage can calcify physiologically, pathological calcification occurs in the setting of systemic diseases, including chronic renal failure, cardiovascular diseases, and metabolic disorders, and malignancy such as chondrosarcoma [1]. Despite its prevalence and association with various diseases, costal cartilage calcification remains understudied due to difficulties in its evaluation by conventional imaging modalities such as magnetic resonance imaging (MRI) and ultrasound [2,3]. MRI is limited in analyzing costal cartilage due to motion artifacts during respiration, while ultrasound requires a high level of operator experience for accurate imaging. On the other hand, conventional radiographs and computed tomography (CT) can depict established macroscopic calcification but cannot capture early molecular changes preceding visible mineral deposition, thus limiting CT’s ability to differentiate active calcification from quiescent processes [4].

The clinical significance of costal cartilage calcification extends beyond its role as an age-related phenomenon. Progressive calcification of the costal cartilage reduces chest wall compliance and may contribute to restrictive ventilatory impairment in older adults, although this relationship remains incompletely characterized [5]. Moreover, premature or accelerated costal cartilage calcification has been observed in a range of systemic conditions, including cardiovascular disease, chronic kidney disease, thyroid dysfunction, and type 2 diabetes mellitus, suggesting that cartilage calcification may serve as a surrogate marker of broader metabolic and vascular dysfunction [5,6,7]. The evaluation of costal cartilage quality is also clinically relevant in preoperative planning for reconstructive procedures, such as rhinoplasty and auricular reconstruction, where calcified cartilage poses challenges for graft manipulation and may compromise surgical outcomes [3,8]. Despite this clinical relevance, a standardized, quantitative imaging approach for assessing costal cartilage calcification at the molecular level has yet to be established, a notable gap in the literature.

Instead, imaging modalities that can detect early molecular changes may be better suited to examine costal cartilage calcification. For instance, ^18^F-sodium fluoride–positron emission tomography/computed tomography (^18^F-NaF-PET/CT) detects the uptake of sodium fluoride-18 in the hydroxyapatite crystals of osseous matrix and allows direct visualization and measurement of bone metabolism. While ^18^F-NaF-PET/CT is routinely used to evaluate metastatic bone disease and osteosarcoma [4,9], its utility in non-oncological diseases such as osteoporosis and spinal degeneration has been increasingly demonstrated [10,11,12,13,14,15,16]. Furthermore, extraosseous uptake in calcifications has been detected in several ^18^F-NaF studies as well, highlighting its sensitivity in detecting molecular changes in structures beyond bone [17,18,19,20,21,22].

In the context of cartilage, the biological basis of ^18^F-NaF uptake is thought to be related to hydroxyapatite deposition within the cartilaginous matrix. As chondrocytes undergo hypertrophy or apoptosis during aging or disease, calcium phosphate crystals, including hydroxyapatite, are deposited in the extracellular matrix. Since ^18^F-NaF preferentially binds to exposed hydroxyapatite surfaces, ^18^F-NaF uptake in cartilage may plausibly reflect ongoing mineralization processes at the molecular level, analogous to the mechanism in bone tissue [7]. However, histological validation of this mechanism in costal cartilage has not been performed, and the precise relationship between tracer uptake intensity and the stage or activity of calcification remains to be established. Accordingly, interpretations of 18F-NaF uptake as evidence of “active” mineralization should be considered inferential rather than demonstrated.

Importantly, costal cartilage calcification is not merely an age-related phenomenon but is associated with systemic pathological processes. An association between quantified costal cartilage calcification volume and markers of long-term glucose exposure (fasting blood glucose and HbA1c) has been reported [6]. Furthermore, coronary artery calcification (CAC), a well-established marker of atherosclerotic burden, may share common systemic drivers with cartilage calcification, as both involve ectopic mineral deposition in soft tissues. Establishing normal age- and sex-dependent reference values for costal cartilage ^18^F-NaF uptake is therefore a prerequisite to identify pathological deviations that may signal underlying cardiovascular or metabolic disease.

In this study, we examine the utility of ^18^F-NaF-PET/CT in the detection of molecular calcification associated with costal cartilage using data from the Cardiovascular Molecular Calcification Assessed by ^18^F-NaF PET/CT (CAMONA) clinical trial. Specifically, we investigate whether ^18^F-NaF uptake in the costal cartilage increases with age, if this relationship differs between males and females, if molecular tracer uptake corresponds to CT-based measures of calcification, and how costal cartilage calcification relates to coronary artery calcification. We hypothesize that ^18^F-NaF uptake in the costal cartilage increases with aging, given that age is a major factor in cartilage calcification, and that this age-related increase differs between males and females.

## 2. Materials and Methods

### 2.1. Subjects

**Figure 1:**
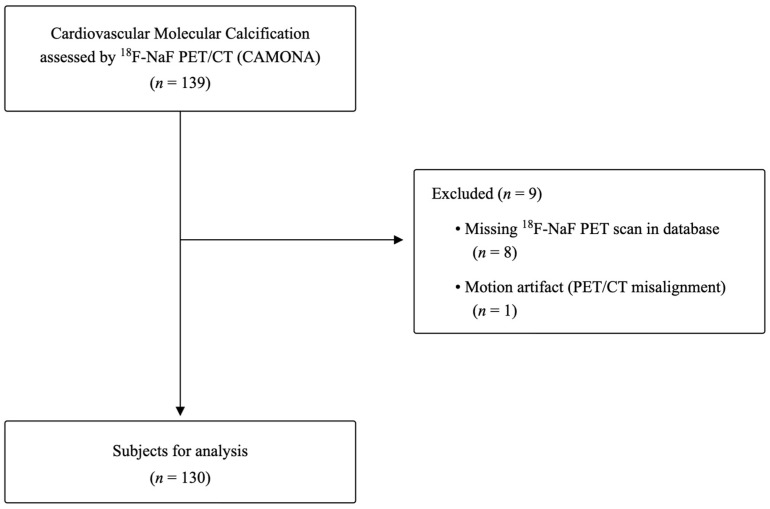
Flow Diagram detailing criteria of participant inclusion and exclusion. Subjects were included based on availability of data and scans in our personal database from the CAMONA study, followed by quality of alignment between PET and CT scans.

This study analyzes ^18^F-NaF PET/CT scans obtained from the CAMONA study (NCT01724749), which was conducted at Odense University Hospital in Denmark and was approved by the Danish National Committee on Biomedical Research Ethics and abided by the Declaration of Helsinki. Further details of the study are described by Blomberg et al. [23]. The CAMONA study population has been previously utilized to assess musculoskeletal metabolism across multiple anatomical sites, including the glenohumeral joint, iliac bones, and arm muscles [10,11,24]. Eight subjects from the original study were excluded due to lack of available ^18^F-NaF scan data in our database, while one additional subject was excluded due to motion artifact that resulted in misalignment between PET and CT scans (Figure 1). Overall, 130 subjects aged 21 to 75 years were included in the analysis.

### 2.2. Image Acquisition

Integrated PET/CT scanners (Discovery 690/710, STE, VCT and RX; GE Healthcare, Chicago, IL, USA) were used to perform ^18^F-NaF PET/CT scans with a protocol previously outlined by Blomberg et al. [25]. Briefly, PET scans were acquired 90 min after intravenous administration of 2.2 MBq of ^18^F-NaF per kilogram of body weight. The dosage varied from 108.7 MBq to 348.1 MBq, depending on the individual’s weight (kg). The mean dosage administered was 174.5 MBq (±36.4). Attenuation, scatter, random coincidence, and scanner dead time corrections were performed. The imaging protocol was constructed in accordance with the practice guidelines of the Society of Nuclear Medicine [26].

### 2.3. Quantitative Image Analysis

**Figure 2:**
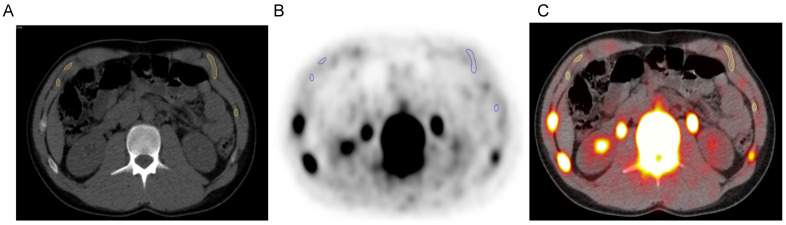
Regions of interest (ROIs) in the costal cartilage. Representative image of the ROI delineated on the axial (**A**) CT, (**B**) PET, and (**C**) fused PET/CT images. The outline of the cartilage is highlighted by the yellow or blue contours, drawn using manually generated CT-based segmentations.

Fused PET/CT images were analyzed using OsiriX software version 2.0 (Pixmeo, Bernex, Switzerland). The region of interest (ROI) was defined as the costal cartilage of ribs 8 to 10 on the left side, with the upper boundary being the cartilage preceding rib 8 and the lower boundary being the cartilage trailing after rib 10 (Figure 2). Specifically, costal cartilages from ribs 8 to 10 were chosen to avoid including osseous tracer uptake from the ribs and sternum and allow for maintenance of a minimum distance of 0.5 to 1.0 cm away from the rib. Additionally, the utilized ROI provides relatively well-defined cartilage area that may be identified consistently across subjects in the cohort. ROI segmentations were constructed manually on axial plane images by tracing the perimeter of the cartilage on the CT images. Mean Hounsfield Unit (HU) measurements were derived from the co-registered CT scans used for attenuation correction of PET images with the same ROI used for the calculation of the mean ^18^F-NaF uptake. For baseline demographic comparisons, HU was calculated as a simple average across slices; for all correlation and age-stratified analyses, area-weighted mean HU was used for consistency with the area-weighted mean standardized uptake value (SUV_mean_).

### 2.4. Statistical Analysis

All statistical analyses were performed using GraphPad Prism 8 (San Diego, CA, USA) and Python 3.12 with SciPy 1.12 (scipy.org). Shapiro–Wilk tests confirmed non-normal distributions for SUV_mean_ and HU values in both males and females (*p* < 0.001); therefore, non-parametric tests were used throughout. Continuous variables are presented as mean ± standard deviation (SD). Spearman’s rank correlation coefficient (r) was used to assess relationships between NaF uptake and age, HU, coronary artery calcium score, and BMI. Spearman correlation was chosen as the primary method for the CAC analysis given the highly skewed, zero-inflated distribution of CAC scores in this cohort (72% with CAC = 0), which may substantially influence parametric correlation estimates. Mann–Whitney U tests compared continuous variables between groups. Kruskal–Wallis tests evaluated differences across age groups (21–29, 30–39, 40–49, 50–59, and 60–75 years), with Dunn’s post hoc test (Bonferroni correction) following significant results. Linear regression derived R^2^ values and 95% confidence intervals (CI); R^2^ values are reported alongside Spearman correlations to provide complementary information, as Spearman ρ assesses monotonic rank-order associations without assuming linearity, while R^2^ quantifies the proportion of variance explained under a linear model. Spearman correlation remains the primary inferential statistic throughout. Fisher’s z-transformation compared correlation coefficients between sexes. Receiver operating characteristic (ROC) analysis evaluated SUV_mean_ discriminative ability for age ≥ 50 years. Fisher’s exact test was used for categorical comparisons. Multiple linear regression included age, sex, and BMI as predictors. Effect sizes were estimated using Cohen’s d. A *p*-value less than 0.05 was considered statistically significant.

## 3. Results

### 3.1. Baseline Characteristics

In total, 130 subjects (mean age 48.7 ± 14.5 years; mean BMI 27.0 ± 4.4 kg/m^2^) were analyzed, comprising 67 females (mean age 50.4 ± 14.6 years; BMI 25.8 ± 3.6 kg/m^2^) and 63 males (mean age 46.9 ± 14.3 years; BMI 28.3 ± 4.9 kg/m^2^). Baseline demographic, clinical, and laboratory characteristics are presented in Table 1. There were no significant differences between sexes in age (*p* = 0.14), SUV_mean_ (*p* = 0.12, Cohen’s d = 0.19), or mean HU (*p* = 0.43, Cohen’s d = 0.12) by Mann–Whitney U test, though BMI was significantly higher in males (*p* = 0.002). In females, the mean ^18^F-NaF uptake was 0.69 ± 0.38 while the corresponding mean HU was 108.0 ± 40.0. In males, the mean ^18^F-NaF uptake was 0.63 ± 0.22, and the mean HU was 104.0 ± 24.0.

### 3.2. Relationship Between ^18^F-NaF and Age

**Figure 3:**
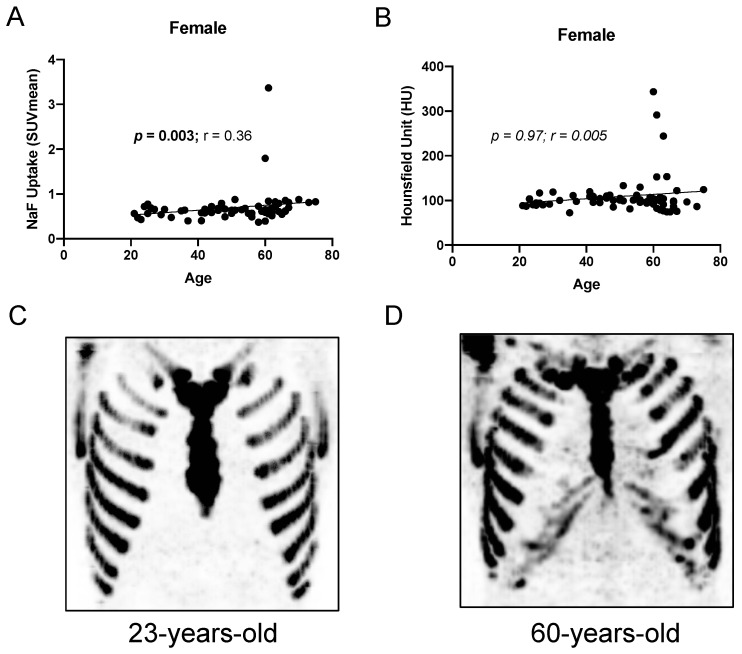
^18^F-NaF uptake in the costal cartilage increases with aging in females. Correlation between aging and (**A**) ^18^F-NaF uptake and (**B**) mean Hounsfield Unit. (**C**,**D**) Representative images of ^18^F-NaF uptake in the costal cartilage of (**C**) a 23-year-old subject and (**D**) an older 60-year-old subject.

**Figure 4:**
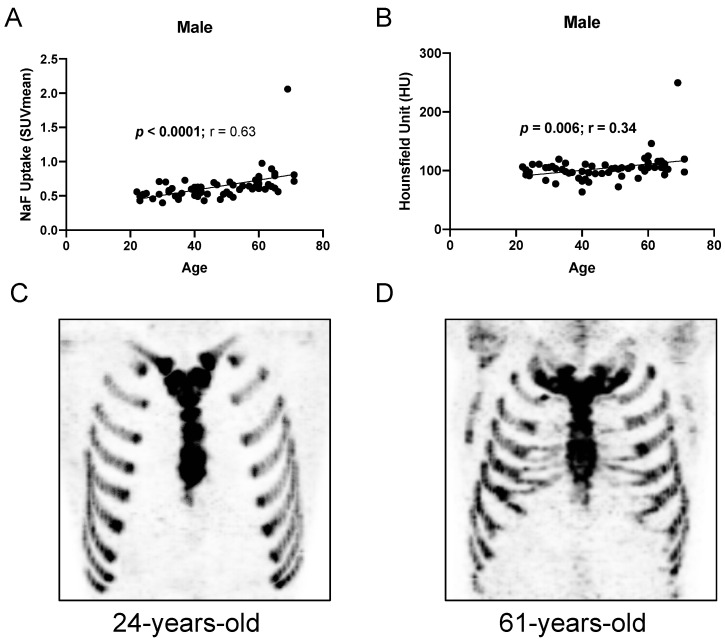
^18^F-NaF uptake in the costal cartilage is increased in older males. There is a positive correlation between age and (**A**) ^18^F-NaF uptake and (**B**) mean Hounsfield Unit in males. (**C**,**D**) Representative images of ^18^F-NaF uptake in the costal cartilage of (**C**) a 24-year-old subject and (**D**) an older 61-year-old subject.

When both sexes were combined, there was a significant overall correlation between costal cartilage ^18^F-NaF uptake and age (Spearman r = 0.50, *p* < 0.0001; linear regression R^2^ = 0.09; Table 2). When stratified by sex, there was a significant correlation between ^18^F-NaF uptake and age in both females (*p* = 0.003, r = 0.36; Figure 3) and males (*p* < 0.0001, r = 0.63; Figure 4). Linear regression yielded R^2^ values of 0.044 (females; slope 95% CI: −0.001 to 0.012) and 0.240 (males; slope 95% CI: 0.004 to 0.011), indicating that age explained a modest, but greater, proportion of variance in SUV_mean_ among males. The association between age and ^18^F-NaF uptake in females and males is illustrated in Figure 3 and Figure 4, respectively. A detailed summary of correlation analysis is provided in Table 2.

### 3.3. Relationship Between PET Uptake and CT Attenuation

There was also a significant correlation between mean HU and age in males (r = 0.34, *p* = 0.006), but not in females (r = 0.005, *p* = 0.97). SUV_max_ also correlated significantly with age in both females (r = 0.28, *p* = 0.024) and males (r = 0.39, *p* = 0.002). There was a trend toward correlation between ^18^F-NaF uptake and mean HU in females (r = 0.23, *p* = 0.063) and males (r = 0.20, *p* = 0.116). Using area-weighted values, the SUV_mean_–HU correlation was similarly modest and non-significant in both sexes (females: r = 0.21, *p* = 0.090; males: r = 0.20, *p* = 0.114).

### 3.4. Age-Stratified Analysis

The following age-sex-stratified analyses are exploratory and should be interpreted with caution given the small sample sizes in some strata, particularly the 30–39-year female group (n = 5). When subjects were stratified into five age groups, a clear pattern of increasing SUV_mean_ with advancing age was observed in both sexes. In females, mean SUV_mean_ ranged from 0.605 ± 0.112 in the youngest group (21–29 years) to 0.832 ± 0.574 in the oldest group (60–75 years). In males, the SUV_mean_ increased from 0.528 ± 0.079 to 0.800 ± 0.334 across the same age range. The Kruskal–Wallis test demonstrated a highly significant difference in SUV_mean_ across age groups in males (H = 25.46, *p* < 0.0001), with a trend in females (H = 8.52, *p* = 0.074). Direct comparison of the youngest and oldest tertiles confirmed significantly higher SUV_mean_ in older subjects for both females (*p* = 0.003) and males (*p* < 0.0001).

Of the 130 subjects, 81 were classified as healthy controls and 49 as non-healthy controls. Non-healthy controls demonstrated significantly higher SUV_mean_ (0.690 ± 0.242 vs. 0.648 ± 0.348; *p* = 0.007), though they were also significantly older (57.1 ± 11.5 vs. 43.6 ± 13.9 years). When stratified by sex, this difference was found to be significant only in males (*p* = 0.003), but not in females (*p* = 0.35). BMI did not significantly correlate with SUV_mean_ in females (r = 0.08, *p* = 0.546) but was significantly correlated in males (r = 0.33, *p* = 0.009). The SUV–age correlation remained robust after controlling for BMI (partial r: females = 0.38, *p* = 0.002; males = 0.62, *p* < 0.0001). A comprehensive summary is presented in Table 3.

### 3.5. Association with CAC Score

Coronary artery calcium (CAC) score data were available for all 130 subjects; 94 subjects (53 females, 41 males) had a CAC score of zero and 36 had positive CAC scores. Fisher’s exact test showed a trend toward higher prevalence of positive CAC in males (34.9% vs. 20.9%; odds ratio (OR) = 2.03, *p* = 0.081). A statistically significant Spearman correlation was observed between CAC score and costal cartilage SUV_mean_ in both females (r = 0.26, *p* = 0.036) and males (r = 0.51, *p* < 0.0001). Given that 72% of the cohort had a CAC score of zero, this association should be interpreted with caution and considered exploratory rather than clinically definitive. The mean ROI area was 285.2 ± 294.4 cm^2^ (median: 162.0 cm^2^), with males having significantly larger ROI areas than females (336.2 ± 332.9 vs. 237.4 ± 246.0 cm^2^; *p* = 0.016). ROI area correlated significantly with age in males (r = 0.49, *p* < 0.0001) but not in females (r = 0.08, *p* = 0.52). A comprehensive summary of all correlation analyses is presented in Table 2.

### 3.6. Additional Analyses

Dunn’s post hoc analysis (Bonferroni-corrected) revealed that in males, the 60–75 age group had significantly higher SUV_mean_ than the 21–29 (*p* = 0.002), 30–39 (*p* = 0.007), and 40–49 (*p* = 0.003) groups, while the 50–59 group did not differ significantly from any other group. Fisher’s z-transformation test confirmed that the sex difference in correlation strength was statistically significant (z = −2.06, *p* = 0.040). Multiple linear regression including age, sex, and BMI yielded an adjusted R^2^ of 0.063 (F = 7.12, *p* < 0.001), with age as the sole significant predictor. The modest pooled R^2^ reflects the divergence between sex-stratified relationships (sex-specific R^2^ = 0.044 in females vs. 0.240 in males; Table 2) and the increasing heteroscedasticity of SUV_mean_ with age (see coefficient of variation analysis below), both of which a single pooled linear model cannot fully capture. Receiver operating characteristic analysis for discriminating subjects aged ≥ 50 years yielded an area under the curve (AUC) of 0.737 overall, 0.635 in females, and 0.832 in males. The coefficient of variation for SUV_mean_ increased substantially with age in both sexes: from 18.4% (females 21–29) to 68.9% (females 60–75) and from 14.9% (males 21–29) to 41.7% (males 60–75), reflecting increasing heterogeneity in calcification patterns with aging.

## 4. Discussion

Our study establishes a quantification technique to assess costal cartilage calcification using ^18^F-NaF-PET/CT. The results demonstrated that tracer uptake increases with age in both males and females. Beyond costal cartilage, this approach, with appropriate modifications to ROI placement, could be applied to other cartilaginous structures to measure calcification under both physiological and pathological conditions. Potential applications include quantifying calcification in localized cartilage malignancies such as chondrosarcomas and investigating the relationship between cartilage calcification and systemic diseases that disrupt calcium homeostasis, including chronic renal failure and metabolic disorders.

Costal cartilage calcification can reduce chest wall compliance and may contribute to restrictive respiratory limitations in older adults [5], yet its functional consequences remain poorly characterized. While dedicated ^18^F-NaF-PET/CT imaging solely for costal cartilage assessment is not currently part of routine clinical practice, opportunistic sub-analysis in patients undergoing ^18^F-NaF-PET/CT for oncology-related indications, who also present with chest pain or stiffness, may help clarify the clinical significance of cartilage uptake, particularly as normative patterns of physiological and pathological uptake become better defined.

A notable finding in this study was the sex-specific difference in the strength of the association between ^18^F-NaF uptake and aging. As detailed in Table 2, males demonstrated a substantially stronger correlation with age than females, and the sex difference in the strength of the ^18^F-NaF uptake–age correlation was statistically significant (Table 2). This sex-based disparity is consistent with prior CT-based studies reporting different calcification morphologies between sexes [1]. The greater variability in SUV_mean_ among older females (CV = 68.9% vs. 41.7% in males) may reflect more heterogeneous calcification patterns. Importantly, the SUV–age correlation remained robust after controlling for BMI, and multiple regression confirmed age as the sole significant predictor (Table 2). The biological mechanisms underlying these sex-specific differences remain speculative and were not directly tested in this study. Possible contributing factors include biological differences in hormonal milieu, particularly the effects of estrogen on cartilage metabolism, as well as sex-dependent variations in body composition and biomechanical loading of the chest wall. These hypotheses warrant dedicated investigation in future studies designed to evaluate hormonal and metabolic covariates.

The dissociation between SUV_mean_ and CT-derived HU values is particularly noteworthy. Despite both metrics correlating with age in males, their mutual correlation was modest and non-significant in both sexes (r = 0.20 in males, r = 0.21 in females using area-weighted values), suggesting that ^18^F-NaF uptake may reflect a component of the calcification process not fully captured by CT attenuation, although this interpretation remains speculative in the absence of histological correlation. The lack of a significant correlation between SUVmean and HU does not invalidate the methodology; rather, it may indicate that PET and CT provide complementary rather than redundant information about cartilage calcification. However, alternative explanations, including partial volume effects, ROI heterogeneity, and noise in HU measurements, cannot be excluded. The observed correlation between CAC scores and costal cartilage SUV_mean_ (r = 0.26 in females, r = 0.51 in males) may indicate that cartilage and vascular calcification share some common systemic drivers, although the underlying mechanisms likely differ and any clinical inference should be considered preliminary. However, this association should be interpreted with caution given the highly skewed, zero-inflated distribution of CAC scores in this cohort (72% with CAC = 0), which may substantially influence correlation estimates regardless of the statistical method used. The near-zero Pearson correlation in females (r = −0.005) compared to the significant Spearman correlation (ρ = 0.257) illustrates the sensitivity of the result to analytic approach, underscoring the need for replication in cohorts with more evenly distributed CAC values.

The ROC analysis demonstrated moderate-to-good discriminative ability to identify subjects aged ≥ 50 years, particularly in males (AUC = 0.832 vs. 0.635 in females). Dunn’s post hoc analysis showing acceleration in the 60–75 age group suggests a non-linear trajectory with a threshold effect around the sixth decade. The increasing coefficient of variation with age underscores heterogeneity in older individuals. Multiple regression indicated age was the only significant predictor in a model including sex and BMI, highlighting substantial unexplained variance likely related to genetic, hormonal, or metabolic factors.

Mechanistically, the increased ^18^F-NaF uptake may correspond to ectopic calcification and calcium crystal deposition within the cartilage associated with aging [7,13,16]. Calcium crystals, such as basic calcium phosphate (BCP) and calcium pyrophosphate dihydrate (CPPD) crystals, are formed due to biological processes involving chondrocytes undergoing hypertrophy, mitophagy, or apoptosis in response to inflammation and aging [7]. Similarly, increased ^18^F-NaF uptake associated with aging and degenerative changes in the lumbar and cervical spine has been previously demonstrated [12,13,15,16]. Interestingly, we only observed a trend, not a significant correlation, between ^18^F-NaF uptake and mean HU, even in males, despite the fact that both significantly correlated with age. One possible explanation is that ^18^F-NaF uptake within the cartilage may precede the formation of macroscopic calcification capable of being detected by CT, or that there is a lack of ^18^F-NaF uptake in completed calcification.

A speculative, but potentially interesting, future application of ^18^F-NaF-PET/CT in the clinical setting may be in the evaluation of cartilage calcification for pre-operative procedures that use costal cartilage grafts, such as rhinoplasty and ear reconstruction [8]. The successful outcome of costal cartilage grafting for auricular reconstruction relies on several factors, such as the strength and size of the available rib cartilage and the presence of adequate healthy tissue with favorable blood supply in the auricular region. Age-related calcification and the resulting stiffness of the cartilage can make it challenging to manipulate the cartilage, leading to unexpected absorption and suboptimal surgical outcomes. In fact, calcification in the costal cartilage and the resulting stiffness has been described to be associated with surgical challenges during graft manipulation [8]. As such, preoperative ultrasonographic examinations have been increasingly used to predict cartilage quality prior to intraoperative harvesting [3]. Similarly, ^18^F-NaF values may provide valuable insight if they were to be associated with graft success.

The pattern of calcification (i.e., central, peripheral, and diffuse) could also be critical in establishing the association of costal cartilage calcification with certain disease processes [1]. For instance, diffuse enlargement of the costochondral junction is associated with acromegaly, while expansile mass is associated with chondrosarcoma [1]. Additionally, heavy premature costal cartilage calcification has been associated with systemic conditions, including malignancy, autoimmune disorders, chronic renal failure, and thyroid disease [5]. Recognizing and properly interpreting radiologic features of the costal cartilage aid in the diagnosis of various systemic conditions and can improve patient outcomes. Although the current study did not investigate the different patterns of calcification or uptake of ^18^F-NaF, it will be important for future research endeavors to explore these factors to compare the patterns and extent of calcification with histological changes in the cartilaginous matrix. In addition to the pattern of calcification, costal cartilage calcification volume has been associated with increased risk for metabolic disorders such as type 2 diabetes mellitus. One study reported that greater quantified costal cartilage calcification was associated with rising fasting blood glucose and HbA1c in female participants after adjustment for age, race, BMI, and glomerular filtration rate, even in females with a coronary artery calcium score of zero [6].

Our study has several limitations. First, we lacked relevant medical or trauma-related history of the subjects. It is possible that some of the subjects demonstrated high ^18^F-NaF values or mean HU in their costal cartilage due to underlying illness or previous injury. Future ^18^F-NaF PET/CT studies should ideally stratify or enroll patients with injuries and diseases to better characterize their impact on calcification of the costal cartilage. Establishing clear correlations between relevant diseases and costal cartilage calcification may delineate age-related changes from disease processes. Second, several age-sex strata contained relatively small sample sizes, which may limit statistical power for subgroup analysis. However, we emphasize that the age-sex-stratified analysis served as an exploratory investigation, aiming to determine potential differences rather than stating definitive age and sex-specific effects. Future studies with larger and more balanced patient cohorts are warranted to further characterize these age and sex-dependent differences. Third, the ROI was manually delineated over costal cartilage of ribs 8-10 on the left side only as we aimed to minimize the influence of adjacent osseous structures (ribs and sternum) on ^18^F-NaF uptake, which could serve as potential confounders, and to facilitate consistent identification across patients. However, restricting the analysis to a subset of costal cartilage may limit full representation of global cartilage calcification. Additionally, while BMI did not confound the SUV–age relationship, future studies would benefit from multivariate regression models that adjust for blood pressure, fasting glucose, lipid profiles, renal function markers, and other potential confounders that may influence calcification processes. Importantly, the CAMONA dataset did not provide individual-level access to all laboratory values (e.g., fasting glucose, lipid profiles, renal function, inflammatory markers), as these were derived from the CAMONA source table rather than from our raw data, precluding comprehensive multivariate adjustment for metabolic confounders. The inclusion of both healthy and non-healthy controls may introduce additional heterogeneity, and although we report subgroup comparisons, the age difference between groups confounds interpretation of health-status effects. Manual ROI segmentation was performed using a standardized protocol by trained radiologists; however, formal interobserver and intraobserver concordance analysis was not conducted, which limits assessment of measurement reproducibility. Future studies should incorporate such analysis to validate the reliability of this quantification technique and may also employ semi-automated methods to reduce operator dependence. The use of AI-based segmentation tools, such as TotalSegmentator, may reduce operator variability and improve reproducibility of ROI measurements [27]. Finally, the cross-sectional design precludes conclusions about temporal progression; longitudinal studies would provide stronger evidence for monitoring cartilage calcification progression.

## 5. Conclusions

The principal contribution of this study is a quantification approach based on ^18^F-NaF-PET/CT for the assessment of costal cartilage calcification. Applying this technique, we observed significantly increased ^18^F-NaF uptake with aging in both females and males. The stronger correlation in males, the dissociation between SUV_mean_ and CT attenuation, and the association with coronary artery calcification collectively raise the hypothesis that molecular imaging may be sensitive to an earlier stage of the calcification process compared to CT, though this interpretation remains to be confirmed by histological and longitudinal studies. Further studies using ^18^F-NaF-PET to correlate ^18^F-NaF uptake in costal cartilage with progression of various pathological conditions may be instrumental in determining whether costal cartilage calcification can serve as a potential biomarker for systemic cardiovascular and metabolic disease risk. These findings establish a methodological framework for future studies incorporating broader costal cartilage segmentation, metabolic parameters, and clinical covariates to better define the biological and clinical significance of costal calcification.

## Figures and Tables

**Table 1:** Demographic, laboratory, and imaging characteristics.

Characteristic	All (n = 130)	Female (n = 67)	Male (n = 63)	*p*-Value
Demographics				
Age (years)	48.7 ± 14.5	50.4 ± 14.6	46.9 ± 14.3	0.144 ^a^
Age range	21–75	21–75	22–71	—
BMI (kg/m^2^)	27.0 ± 4.4	25.8 ± 3.6	28.3 ± 4.9	0.002 ^a^
Systolic BP (mmHg)	128.1 ± 16.1	126.2 ± 18.2	129.9 ± 13.6	†
Diastolic BP (mmHg)	76.9 ± 9.1	76.2 ± 9.8	77.7 ± 8.3	†
Healthy controls, n (%)	81 (62.3%)	42 (62.7%)	39 (61.9%)	1.000 ^b^
Laboratory				
Fasting glucose (mmol/L)	5.7 ± 0.7	5.6 ± 0.4	5.7 ± 0.8	†
HbA1c (mmol/L)	35.0 ± 4.8	34.3 ± 4.0	35.8 ± 5.5	†
Fibrinogen (µmol/L)	10.7 ± 9.0	10.6 ± 9.4	10.8 ± 8.7	†
WBC (10^9^/L)	6.1 ± 2.0	6.0 ± 1.7	6.3 ± 2.2	†
Total cholesterol (mmol/L)	5.1 ± 0.9	5.1 ± 0.9	5.1 ± 0.9	†
LDL cholesterol (mmol/L)	3.2 ± 0.8	3.1 ± 0.9	3.2 ± 0.8	†
HDL cholesterol (mmol/L)	1.4 ± 0.5	1.5 ± 0.5	1.4 ± 0.5	†
Triglycerides (mmol/L)	1.13 ± 0.70	1.13 ± 0.70	1.14 ± 0.69	†
Creatinine (µmol/L)	80.2 ± 16.7	78.7 ± 13.9	81.7 ± 19.0	†
eGFR (mL/min/1.73 m^2^)	80.3 ± 14.4	80.9 ± 12.5	79.7 ± 16.2	†
Imaging				
Injected dose (MBq)	174.5 ± 36.4	158.5 ± 25.5	191.7 ± 38.7	<0.0001 ^a^
SUV_mean_	0.66 ± 0.31	0.69 ± 0.38	0.63 ± 0.22	0.115 ^a^
Mean HU	105.9 ± 33.1	107.8 ± 39.7	103.9 ± 24.2	0.428 ^a^
ROI area (cm^2^)	285.2 ± 294.4	237.4 ± 246.0	336.2 ± 332.9	0.016 ^a^
CAC score (AU)	89.6 ± 269.8	47.1 ± 166.8	134.8 ± 343.2	0.060 ^a^
CAC = 0, n (%)	94 (72.3%)	53 (79.1%)	41 (65.1%)	0.081 ^b^

BMI: Body mass index; BP: Blood pressure; CAC: Coronary artery calcium; eGFR: Estimated glomerular filtration rate; HDL: High-density lipoprotein; HU: Hounsfield unit; LDL: Low-density lipoprotein; ROI: Region of interest; SUV_mean_: mean standardized uptake value; WBC: White blood cells. Values are mean ± SD unless noted. SUV_mean_ = area-weighted; HU = simple average (matching CAMONA source). ^a^ Mann–Whitney U. ^b^ Fisher’s exact test. † From CAMONA source table (individual-level lab data not in raw dataset; *p*-values cannot be independently computed).

**Table 2:** Spearman rank correlation matrix.

Spearman ρ	All	Female	Male	Fisher’s z
Uptake vs. Age				
SUV_mean_ vs. Age	0.499 ***	0.358 **	0.632 ***	*p* = 0.040 *
Avg SUV_mean_ vs. Age	0.529 ***	0.423 ***	0.633 ***	*p* = 0.101
SUV_max_ vs. Age	0.340 ***	0.276 *	0.388 **	*p* = 0.483
R^2^ (OLS: SUV_mean_∼Age)	0.093 **	0.044	0.240 ***	—
Partial ρ (adj. BMI)	0.508 ***	0.378 **	0.624 ***	—
CT Density				
HU vs. Age	0.169	0.005	0.341 **	*p* = 0.051
SUV_mean_ vs. HU	0.208	0.208	0.201	*p* = 0.967
Other Variables				
ROI Area vs. Age	0.262 **	0.079	0.493 ***	*p* = 0.010 *
SUV_mean_ vs. BMI	0.165	0.075	0.328 **	*p* = 0.142
SUV_mean_ vs. CAC	0.359 ***	0.257 *	0.514 ***	*p* = 0.089

BMI: Body mass index; CAC: Coronary artery calcium; HU: Hounsfield unit; ROI: Region of interest; SUV_max_: maximum standardized uptake value; SUV_mean_: mean standardized uptake value. Note: For CAC, Spearman ρ = 0.257 (F) and 0.514 (M), but Pearson r = −0.005 (F) and 0.717 (M) due to zero-inflation (72% of patients have CAC = 0). The choice of correlation method substantially affects results for CAC. Spearman is recommended for non-normal data. * *p* < 0.05, ** *p* < 0.01, *** *p* < 0.001. Fisher’s z compares female vs. male coefficients. R^2^ from OLS. Partial ρ: Spearman on BMI-residualized values. SUV_mean_ = area-weighted (Sum(SUV_mean_ × Area)/Sum(Area)); Avg SUV_mean_ = simple (unweighted) average of per-slice SUV_mean_ values; HU = area-weighted.

**Table 3:** Age-stratified data by sex.

Age	Sex	n	SUV_mean_ ± SD	Median	HU ± SD	ROI (cm^2^)
21–29	F	10	0.605 ± 0.112	0.608	95 ± 9	100 ± 41
21–29	M	9	0.528 ± 0.079	0.523	100 ± 9	159 ± 212
30–39	F	5	0.558 ± 0.114	0.620	100 ± 18	266 ± 223
30–39	M	11	0.558 ± 0.101	0.557	101 ± 12	200 ± 302
40–49	F	12	0.615 ± 0.102	0.620	104 ± 9	164 ± 93
40–49	M	14	0.561 ± 0.079	0.558	94 ± 13	523 ± 455
50–59	F	14	0.610 ± 0.116	0.612	104 ± 14	276 ± 272
50–59	M	11	0.616 ± 0.075	0.636	100 ± 13	281 ± 164
60–75	F	26	0.832 ± 0.574	0.705	121 ± 68	298 ± 307
60–75	M	18	0.800 ± 0.334	0.731	119 ± 35	396 ± 304
KW p	F	—	0.074	—	0.225	0.191
KW p	M	—	<0.0001	—	0.004	0.0002

HU: Hounsfield unit; ROI: Region of interest; SD: Standard deviation; SUV_mean_: mean standardized uptake value. HU values use area-weighted mean (matching original manuscript Table 1 format from prior publication). KW = Kruskal–Wallis across 5 age groups. Dunn’s post hoc (Bonferroni, 10 comparisons) for male SUV_mean_: 21–29 vs. 60–75 p_aej_ = 0.002, 30–39 vs. 60–75 p_aej_ = 0.007, 40–49 vs. 60–75 p_aej_ = 0.003. All other pairs NS after correction.

## Data Availability

The original contributions presented in this study are included in the article. Further inquiries can be directed to the corresponding author.
